# Endotracheal Intubation Versus No Endotracheal Intubation During Cardiopulmonary Arrest in the Emergency Department

**DOI:** 10.7759/cureus.19760

**Published:** 2021-11-20

**Authors:** Abdullah Bakhsh, Reema Alghoribi, Rehab Arbaeyan, Raghad Mahmoud, Sana Alghamdi, Shahd Saddeeg

**Affiliations:** 1 Department of Emergency Medicine, King Abdulaziz University Hospital, Jeddah, SAU

**Keywords:** rosc, outcomes of emergency department cpr, endotracheal intubation in cpr, emergency department cpr, airway in cpr

## Abstract

Background

There is a lack of studies addressing the short and long-term outcomes of using different airway interventions in patients with cardiopulmonary arrest in the emergency department (ED). This retrospective chart review aimed to investigate the effect of endotracheal intubation (ETI) versus no ETI during cardiopulmonary arrest in the ED on return of spontaneous circulation (ROSC) and survival to discharge.

Methodology

A total of 168 charts were reviewed from August 2017 to April 2019. Resuscitation characteristics were obtained from Utstein-style-based cardiopulmonary arrest flow sheets.

Results

Unadjusted analysis showed no difference in ROSC (45.5% in ETI vs. 54.5% in no-ETI) (p = 0.08) and survival to hospital discharge at 28 days (26.7% in ETI vs. 73.3% in non-ETI) (p = 0.07) when comparing ETI versus non-ETI airway management methods during cardiopulmonary resuscitation (CPR). After adjusting for confounding factors, our regression analysis revealed that the use of ETI is associated with lower odds of ROSC (odds ratio [OR] = 3.40, 95% confidence interval [CI] = [0.14-0.84]) and survival to hospital discharge at 28 days (OR = 0.20, 95% CI = [0.04-0.84]).

Conclusions

ETI during CPR in the ED is associated with worse ROSC and survival to hospital discharge at 28 days.

## Introduction

Most patients who experience cardiopulmonary arrest die during the event despite advances in resuscitation science. Those who do achieve return of spontaneous circulation (ROSC) have low survival to discharge rates and poor neurologic outcomes [1,2]. Current guidelines prioritize receiving high-quality chest compressions with minimal interruptions and defibrillation over airway intervention or drug administration [3,4]. The effects of airway intervention techniques used during cardiopulmonary resuscitation (CPR) are not yet fully understood. There is no high-quality evidence favoring one approach for oxygenation and ventilation during CPR [3,5]. The 2020 American Heart Association (AHA) guidelines allow either a standard bag-valve mask (BVM) or an advanced airway device such as an endotracheal tube (ETT) or a supraglottic airway device (SGA) based on the skills and experience of the provider in in-hospital settings [3].

Endotracheal intubation (ETI) remains the most commonly used airway intervention during in-hospital cardiopulmonary arrests [6,7]. Advanced airway procedures should be attempted by experienced and skilled providers to avoid prolonged interruptions of chest compressions and to delay defibrillation [3,5]. Advanced airway techniques in the out-of-hospital setting have been associated with increased hands-off time and hyperventilation, causing decreased coronary perfusion pressures (in animal studies) and increased mortality [8-13]. Although the use of BVM and laryngeal mask airway (LMA) is simple and quick in emergencies and requires minimal training, these devices are used less frequently.

A recently published meta-analysis showed better ROSC and survival to hospital discharge rates with ETI use than with BVM and LMA use in out-of-hospital cardiac arrest (OHCA) patients [14]. Another recently published randomized clinical trial on OHCA patients concluded that favorable neurologic status at discharge, survival to hospital discharge, and ROSC on arriving at the emergency department (ED) were in favor of laryngeal tube insertion over ETI [15]. Moreover, a randomized controlled trial (RCT) in a similar setting showed no significant favorable functional outcome at 30 days between SGA and ETI [16]. A large observational study of in-hospital cardiac arrest (IHCA) patients demonstrated that early ETI, within 15 minutes of the arrest, does not improve survival [6].

Published work focuses on the out-of-hospital setting with controversial results, with only a few focusing on the in-hospital setting. It is important to note the difference in provider expertise between in- and out-of-hospital settings. Additionally, the etiological factors are vastly different between the two settings, potentially affecting the outcomes. The ED is a unique setting with round-the-clock availability of highly skilled providers. There is a lack of studies addressing airway intervention techniques on the outcomes of patients with cardiopulmonary arrest in the ED. Hence, we aimed to evaluate the effect of ETI versus no ETI during CPR in the ED on sustained ROSC and survival to hospital discharge.

## Materials and methods

Study design and population

This retrospective chart review was conducted at the ED of a teaching hospital with an approximate emergency department visit rate of 66,000 per year. After obtaining institutional review board (IRB) approval (156-19), all charts of patients with cardiopulmonary arrest at the ED from August 2017 to April 2019 were reviewed. The ED treated a total of 249 patients with cardiopulmonary arrests during the study period. Of those only 168 met the inclusion criteria. We included all patients who suffered cardiopulmonary arrests in the ED within 12 hours of triage time (this was selected based on the standard ED boarding time), those aged ≥18 years, those receiving intravenous epinephrine within three minutes of recognition, those with non-shockable rhythms, and those without prior advanced airway in place. We excluded charts of patients with a do-not-resuscitate (DNR) order, pre-hospital cardiopulmonary arrest, a prior advanced airway in place, pregnancy, and traumatic arrest. The most common rhythm in our institution’s ED is non-shockable. Patients with shockable rhythms were excluded because the prognosis is different between shockable and non-shockable rhythms, which can be a source of confounding. In addition, we wanted to study the association with a specific patient population.

Study endpoints

Our primary endpoint was sustained ROSC which is defined as the restoration of palpable carotid pulse for at least 20 minutes [17]. Our secondary endpoint was survival to hospital discharge at 28 days.

Study setting

Cardiopulmonary arrests identified in the ED were run via standard advanced cardiac life support protocol. ETIs were performed by emergency medicine residents or board-certified emergency medicine physicians. The recommended method for intubation in the department is video laryngoscopy using a GlideScope® video monitor with a titanium blade (Verathon Inc., WA, USA). Healthcare providers in the ED were required to maintain valid certification in advanced cardiac life support. An arrest flow sheet was completed in real-time by a designated nurse documenting details and times. None of the patients were placed on targeted temperature management protocol post-cardiac arrest in the ED due to a lack of cooling devices. Rather, a fever-control approach was adopted to target a rectal temperature less than 37.9°C. All cardiopulmonary arrests (including boarding patients) in the ED were led by emergency physicians. Boarding patients typically stay in the ED for up to 12 hours due to the lack of in-patient beds. However, this is subject to change based on the inpatient capacity and workforce.

Data collection

Study investigators collected data directly from cardiopulmonary arrest flowsheets. Thereafter, collected data were reviewed by the principal investigator for confirmation. For patients with multiple cardiopulmonary arrests, only the first episode was included (all patients surviving the first episode of CPR had endotracheal tubes placed and, therefore, subsequent episodes met the exclusion criteria). The flowsheet used was standard for all cardiopulmonary arrest patients in the hospital who required chest compressions. It was completed in real-time by a nurse trained in advanced cardiac arrest life support documentation. The presence of sustained ROSC was determined from the cardiopulmonary arrest flowsheet. Survival to discharge at 28 days was obtained from electronic medical records (Phoenix health information system).

Statistical analysis

Frequencies and percentages were used to express categorical data such as gender, diagnosis at admission, time of cardiac arrest, type of airway used (ETI vs. non-ETI), initial rhythm (pulseless electrical activity [PEA] vs. asystole), sustained ROSC, and survival to discharge. Means and standard deviations were used to express continuous data such as age and duration of CPR. Pearson’s chi-square test or independent t-test was used to compare variables between the ETI and the non-ETI group. Moreover, we constructed a logistic regression model for analyzing the primary outcome of ROSC as an independent categorical variable according to patients receiving ETI versus no ETI during CPR to determine potential associations between confounding variables, including age, gender, initial rhythm (PEA/asystole), CPR duration, CPR time (day/night), and diagnosis. The selection of these variables was based on previous studies. A p-value of <0.05 was considered statistically significant. We report unadjusted and adjusted odds ratios (ORs) with 95% confidence intervals (CIs). Analysis was performed using SPSS version 25.0 (IBM Corp., Armonk, NY, USA).

## Results

We reviewed 249 charts of adult patients with cardiopulmonary arrest in the ED. A total of 81 patients were excluded for not meeting the inclusion criteria. A total of 168 patients were included in the study. Of the included patients, 98 (58.3%) were males, and the mean age of the study population was 59 ± 15 years. PEA was seen in 99 (58.9%) patients. The mean time of CPR duration was 18.8 ± 11.7 minutes. ETI was the most common method used during resuscitation; it was used in 87 (51.8%) patients. The mean time to airway intervention was 7.16 ± 6.03 minutes. ETI was not performed in 81 (41.1%) patients. Sustained ROSC was achieved in 88 (52.4%) patients. Of those achieving sustained ROSC, only 15 (17.0%) survived to hospital discharge. Patients with a cardiac diagnosis had a higher rate of non-ETI airway intervention (p < 0.01), whereas patients with a central nervous system diagnosis had higher rates of ETI airway interventions (p = 0.01). Table 1 provides further details on overall study demographics, resuscitation characteristics, and outcomes.

**Table 1:** Patient demographics, resuscitation characteristics, and outcomes. ETI: endotracheal intubation; CPR: cardiopulmonary resuscitation; ROSC: return of spontaneous circulation

	n = 168
Age, mean (SD), years	59.6 ± 15
Sex, n (%)	
Males	98 (58.3%)
Females	70 (41.7%)
Diagnosis at admission, n (%)	
Cardiac	73 (43.5%)
Respiratory disease	47 (28.0%)
Metabolic	22 (13.1%)
Central nervous system	16 (9.5%)
Gastrointestinal	10 (6.0%)
Time of cardiac arrest, n (%)	
Day, 7:00 AM to 10:59 PM	108 (64.3%)
Night, 11:00 PM to 6:59 AM	60 (35.7%)
Duration of CPR, mean (SD), minutes	18.8 (11.7)
Initial rhythm, n (%)	
Pulseless electrical activity	99 (58.9%)
Asystole	69 (41.1%)
Type of airway, n (%)	
ETI	87 (51.8%)
Non-ETI	81 (48.2%)
Mean time of airway intervention, mean (SD), minutes	7.16 (6.03)
Outcomes	
ROSC, n (%)	
Yes	88 (52.4%)
No	80 (47.6%)
Survival to discharge, n (%)	n = 88
Yes	15 (17.0%)
No	73 (82.9%)

When comparing outcomes in the use of ETI versus non-ETI, the groups were statistically similar, as seen in Table 2. Sustained ROSC was slightly more frequent in the non-ETI group; however, these results were not statistically different (45.5% in ETI vs. 54.5% in no-ETI) (p= 0.08). Additionally, survival to hospital discharge rates, although seen more frequently in the non-ETI group results were not statistically different (26.7% in ETI vs. 73.3% in non-ETI) (p= 0.07). 

**Table 2:** Comparison between ETI versus non-ETI. PEA: pulseless electrical activity; ETI: endotracheal intubation; ROSC: return of spontaneous circulation; OR: odds ratio; CI: confidence interval

	ETI	No ETI	P-value	OR (95% CI)
Age	59.28 ± 15.89	59.97 ± 14.87	0.77	--
Gender				
Male	45 (45.9%)	53 (54.1%)	0.07	0.56 (0.30-1.0)
Female	42 (60.0%)	28 (40.0%)		
Diagnosis				
Cardiac	29 (39.7%)	44 (60.3%)	<0.01	--
Respiratory	25 (53.2%)	22 (46.8%)	0.82	--
Metabolic	15 (68.2%)	7 (31.8%)	0.09	--
Central nervous system	13 (81.3%)	3 (18.8%)	0.01	--
Gastrointestinal	5 (50.0%)	5 (50.0%)	0.90	--
Time of arrest				
Day	57 (52.8%)	51 (47.2%)	0.73	1.11 (0.59-2.10)
Night	30 (50.0%)	30 (50.0%)		
Initial rhythm				
PEA	49 (49.5%)	50 (50.5%)	0.47	0.79 (0.42-1.48)
Asystole	38 (55.1%)	31 (44.9%)		
Outcome				
Sustained ROSC	40 (45.5%)	48 (54.5%)	0.08	0.58 (0.31-1.0)
Survival to discharge	4 (26.7%)	11 (73.3%)	0.07	0.30 (0.09-1.00)

When creating a logistic regression model with ROSC as the independent variable, ETI during CPR was 3.4 times more likely to predict no ROSC (OR = 3.40, 95% CI = [0.14-0.84]). Meanwhile, a longer duration of CPR (OR = 1.18, 95% CI = [1.12-1.25]) and asystole (OR = 3.40, 95% CI = [1.46-7.92]) predicted lower likelihood of ROSC (Table 3).

**Table 3:** Adjusted odds ratios for potential confounding factors with ROSC as an independent variable. CPR: CPR: cardiopulmonary resuscitation; ROSC: return of spontaneous circulation; aOR: adjusted odds ratio; CI: confidence interval

	P-value	aOR [95% CI]
Endotracheal intubation	0.01	0.34 [0.14-0.84]
Initial rhythm	<0.01	3.40 [1.46-7.92]
CPR duration	<0.01	1.18 [1.12-1.25]

When selecting survival to hospital discharge as the independent variable, the use of ETI during CPR was 0.20 times more likely to predict lower survival to discharge (OR = 0.20, 95% CI = [0.04-0.84]) (Table 4).

**Table 4:** Adjusted odds ratios for potential confounding factors with survival to hospital discharge as an independent variable. aOR: adjusted odds ratio; CI: confidence interval

	P-value	aOR [95% CI]
Endotracheal intubation	0.02	0.20 [0.04-0.84]

## Discussion

In this retrospective chart review conducted at a single-center ED, we analyzed the outcomes of 168 cardiac arrest patients with documented airway placement during resuscitation. No significant difference was seen in ROSC and survival to discharge rates between the two airway categories (ETI vs. non-ETI). However, when adjusting for potential confounding factors, the use of ETI during CPR was independently associated with lower odds of ROSC and survival to discharge.

The optimal choice of airway intervention is a controversial aspect of resuscitation. Most guidelines are based on data obtained from out-of-hospital settings [3,5]. Previous studies have concluded that the majority of OHCA are unwitnessed, CPR is often delayed, and higher proportions of shockable rhythms are reported [13,18-23]. Conversely, IHCA occurs in monitored settings and is attended to by physicians [24]. IHCA studies have also shown overall better survival outcomes than out-of-hospital settings [25,26]. Therefore, it is challenging to apply the results of OHCA studies to IHCA patients directly.

The goal of CPR is the immediate restoration of the mechanical function of the heart and to improve survival with a good neurological outcome. Several OHCA studies have addressed the effect of different airway interventions on functional outcomes [13,15,16,27-29]. Our analysis confirms an association between ETI during CPR and worse sustained ROSC and survival to hospital discharge. This is consistent with previous results of a large observational study conducted by Hasegawa et al. in Japan on OHCA patients (n = 649,359) [13]. This study found a significant negative association between any advanced airway (ETI or LMA) and all three endpoint measures (OR = 0.38, 95% CI = [0.36-0.39]) for favorable neurologic outcome, (OR = 0.73, 95% CI = [0.71-0.75]) for one month survival, and (OR = 0.67, 95% CI = [0.66-0.69]) for ROSC. This might be explained by the quick establishment of ventilation when using BVM, which reduces the hands-off time leading to successful recovery. Contrary to our result, a recently published RCT by Jabre et al. included 2,043 patients with OHCA [27]. This trial failed to favor BVM versus ETI use for favorable 28-day neurological outcomes. In another RCT in-OHCA setting conducted by Benger et al. investigating functional outcome at discharge or at day 30 for non-discharged patients, a significant difference was not found when comparing SGA to ETI [16].

Andersen et al. recently published an observational cohort study of in-hospital cardiac patients which showed that ETI during the first 15 minutes of resuscitation was associated with reduction in good functional outcomes at hospital discharge compared to those not intubated (10.6% vs. 13.6%; p < 0.001) [6]. ETI can also increase the hands-off time to exceed the recommended time frame for securing the airway [30]. Multiple attempts to intubate by the provider can also occur due to the complexity of the procedure. Additionally, interrupting chest compressions can adversely affect the forward flow of blood to any part of the body, especially to the coronary and cerebral arteries, affecting survival and neurological outcome [31].

Interestingly, a meta-analysis including 13 articles compared the efficacy of BVM, LMA, and ETI in out-of-hospital settings and found that eight studies reporting ROSC between LMA and BVM showed no superiority and based on the pooled analysis of seven studies demonstrated that ETI had a significantly higher ROSC rate than LMA (48% vs. 23%; RR = 0.72; 95% CI = 0.65-0.80; p = 0.01) [14]. This can be attributed to the unfamiliarity with using LMA during OHCA. ETI in a pilot study on human cadavers showed better protection of the airway from regurgitation and aspiration of gastric contents [32], which may play a role in improving the respiratory status of patients.

An indirect comparison was also performed in which no difference was demonstrated between BVM and ETI in improving ROSC, which was corroborated in another study [33]. Yeung et al. published a prospective observational study following both OHCA and IHCA patients (n = 100) and showed that ROSC rates between ETT, LMA, and BVM were not different (48% vs. 25% vs. 32%; p = 0.079) [34]. Conversely, Andersen et al. showed a lower ROSC among tracheal intubated patients than those not intubated [6].

Current literature examining airway management during CPR and its effect on outcomes is controversial. This is probably due to the mulitple confounding factors affecting cardiac arrest outcomes. Our study is unique by including only ED cardiac arrests, therefore, eliminiating inexperienced personnel in airway management during cardiac arrest. Based on our findings, we recommend that clinicans focus on quality chest compressions while using clinical judgment to decide the most appropriate airway device.

Limitations

Because this small study was conducted in a single-center ED, our results may not be generalizable to the entire population. Due to the retrospective nature of the study, unmeasured confounding variables might have influenced our outcomes, such as quality of chest compressions and ventilation, and unrecorded intubation attempts that were not successful. Although no data were available to monitor the quality of CPR, all emergency providers are required to maintain certification in advanced cardiac life support. Our small sample size was limited by the volume of patients in the ED with documented airway placements during CPR throughout the study period. Finally, unaccounted patient management variables during 30 days after a cardiopulmonary arrest could potentially influence the outcomes.

## Conclusions

This small single-center ED study reveals that, after adjusting for potential confounding factors, ETI is associated with lower odds of ROSC and survival to hospital discharge at 28 days. The primary goal during CPR should be to focus on providing high-quality chest compressions while minimizing interruptions. We recommend conducting RCTs with a larger sample size of patients with ED cardiac arrest and prospectively following the patients for a better understanding of how using different airway interventions can influence patient outcomes.

## References

[1] Peberdy MA, Kaye W, Ornato JP (2003). Cardiopulmonary resuscitation of adults in the hospital: a report of 14720 cardiac arrests from the National Registry of Cardiopulmonary Resuscitation. Resuscitation.

[2] Stiell IG, Wells GA, Field B (2004). Advanced cardiac life support in out-of-hospital cardiac arrest. N Engl J Med.

[3] Panchal AR, Bartos JA, Cabañas JG (2020). Part 3: Adult Basic and Advanced Life Support: 2020 American Heart Association Guidelines for Cardiopulmonary Resuscitation and Emergency Cardiovascular Care. Circulation.

[4] Perkins GD, Olasveengen TM, Maconochie I (2018). European Resuscitation Council Guidelines for Resuscitation: 2017 update. Resuscitation.

[5] Soar J, Maconochie I, Wyckoff MH (2019). 2019 international consensus on cardiopulmonary resuscitation and emergency cardiovascular care science with treatment recommendations: summary from the basic life support; advanced life support; pediatric life support; neonatal life support; education, implementation, and teams; and first aid task forces. Circulation.

[6] Andersen LW, Granfeldt A, Callaway CW (2017). Association between tracheal intubation during adult in-hospital cardiac arrest and survival. JAMA.

[7] Kaki AM, Alghalayini KW, Alama MN, Almazroaa AA, Khathlan NA, Sembawa H, Ouseph BM (2017). An audit of in-hospital cardiopulmonary resuscitation in a teaching hospital in Saudi Arabia: a retrospective study. Saudi J Anaesth.

[8] Wang HE, Simeone SJ, Weaver MD, Callaway CW (2009). Interruptions in cardiopulmonary resuscitation from paramedic endotracheal intubation. Ann Emerg Med.

[9] Gaszynski T, Gaszynska E (2013). The influence of different airway management strategies on chest compression fraction in simulated cardiopulmonary resuscitation, provided by paramedics: LMA Supreme versus Endotracheal Intubation and Combitube. Signa Vitae.

[10] Aufderheide TP, Sigurdsson G, Pirrallo RG (2004). Hyperventilation-induced hypotension during cardiopulmonary resuscitation. Circulation.

[11] Aufderheide TP, Lurie KG (2004). Death by hyperventilation: a common and life-threatening problem during cardiopulmonary resuscitation. Crit Care Med.

[12] Fouche PF, Simpson PM, Bendall J, Thomas RE, Cone DC, Doi SA (2014). Airways in out-of-hospital cardiac arrest: systematic review and meta-analysis. Prehosp Emerg Care.

[13] Hasegawa K, Hiraide A, Chang Y, Brown DF (2013). Association of prehospital advanced airway management with neurologic outcome and survival in patients with out-of-hospital cardiac arrest. JAMA.

[14] Yang Z, Liang H, Li J (2019). Comparing the efficacy of bag-valve mask, endotracheal intubation, and laryngeal mask airway for subjects with out-of-hospital cardiac arrest: an indirect meta-analysis. Ann Transl Med.

[15] Wang HE, Schmicker RH, Daya MR (2018). Effect of a strategy of initial laryngeal tube insertion vs endotracheal intubation on 72-hour survival in adults with out-of-hospital cardiac arrest: a randomized clinical trial. JAMA.

[16] Benger JR, Kirby K, Black S (2018). Effect of a strategy of a supraglottic airway device vs tracheal intubation during out-of-hospital cardiac arrest on functional outcome: the AIRWAYS-2 randomized clinical trial. JAMA.

[17] Cummins RO, Chamberlain D, Hazinski MF (1997). Recommended guidelines for reviewing, reporting, and conducting research on in-hospital resuscitation: the in-hospital 'Utstein style'. A statement for healthcare professionals from the American Heart Association, the European Resuscitation Council, the Heart and Stroke Foundation of Canada, the Australian Resuscitation Council, and the Resuscitation Councils of Southern Africa. Resuscitation.

[18] Tanabe S, Ogawa T, Akahane M (2013). Comparison of neurological outcome between tracheal intubation and supraglottic airway device insertion of out-of-hospital cardiac arrest patients: a nationwide, population-based, observational study. J Emerg Med.

[19] McMullan J, Gerecht R, Bonomo J, Robb R, McNally B, Donnelly J, Wang HE (2014). Airway management and out-of-hospital cardiac arrest outcome in the CARES registry. Resuscitation.

[20] Buanes EA, Heltne JK (2014). Comparison of in-hospital and out-of-hospital cardiac arrest outcomes in a Scandinavian community. Acta Anaesthesiol Scand.

[21] Daya MR, Schmicker RH, Zive DM (2015). Out-of-hospital cardiac arrest survival improving over time: results from the Resuscitation Outcomes Consortium (ROC). Resuscitation.

[22] Hasselqvist-Ax I, Riva G, Herlitz J (2015). Early cardiopulmonary resuscitation in out-of-hospital cardiac arrest. N Engl J Med.

[23] Meaney PA, Nadkarni VM, Kern KB, Indik JH, Halperin HR, Berg RA (2010). Rhythms and outcomes of adult in-hospital cardiac arrest. Crit Care Med.

[24] Girotra S, Nallamothu BK, Spertus JA, Li Y, Krumholz HM, Chan PS (2012). Trends in survival after in-hospital cardiac arrest. N Engl J Med.

[25] Sittichanbuncha Y, Prachanukool T, Sawanyawisuth K (2013). A 6-year experience of CPR outcomes in an emergency department in Thailand. Ther Clin Risk Manag.

[26] Fairbanks RJ, Shah MN, Lerner EB, Ilangovan K, Pennington EC, Schneider SM (2007). Epidemiology and outcomes of out-of-hospital cardiac arrest in Rochester, New York. Resuscitation.

[27] Jabre P, Penaloza A, Pinero D (2018). Effect of bag-mask ventilation vs endotracheal intubation during cardiopulmonary resuscitation on neurological outcome after out-of-hospital cardiorespiratory arrest: a randomized clinical trial. JAMA.

[28] Wang HE, Szydlo D, Stouffer JA (2012). Endotracheal intubation versus supraglottic airway insertion in out-of-hospital cardiac arrest. Resuscitation.

[29] Shin SD, Ahn KO, Song KJ, Park CB, Lee EJ (2012). Out-of-hospital airway management and cardiac arrest outcomes: a propensity score matched analysis. Resuscitation.

[30] Gruber C, Nabecker S, Wohlfarth P (2013). Evaluation of airway management associated hands-off time during cardiopulmonary resuscitation: a randomised manikin follow-up study. Scand J Trauma Resusc Emerg Med.

[31] Lurie KG, Nemergut EC, Yannopoulos D, Sweeney M (2016). The physiology of cardiopulmonary resuscitation. Anesth Analg.

[32] Piegeler T, Roessler B, Goliasch G, Fischer H, Schlaepfer M, Lang S, Ruetzler K (2016). Evaluation of six different airway devices regarding regurgitation and pulmonary aspiration during cardio-pulmonary resuscitation (CPR) - a human cadaver pilot study. Resuscitation.

[33] Malinverni S, Bartiaux M, Cavallotto F, De Longueville D, Mols P, Gorlicki J, Adnet F (2019). Does endotracheal intubation increases chest compression fraction in out of hospital cardiac arrest: a substudy of the CAAM trial. Resuscitation.

[34] Yeung J, Chilwan M, Field R, Davies R, Gao F, Perkins GD (2014). The impact of airway management on quality of cardiopulmonary resuscitation: an observational study in patients during cardiac arrest. Resuscitation.

